# Stimulatory Secretions of Airway Epithelial Cells Accelerate Early Repair of Tracheal Epithelium

**DOI:** 10.1038/s41598-017-11992-6

**Published:** 2017-09-15

**Authors:** Egi Kardia, Rafeezul Mohamed, Badrul Hisham Yahaya

**Affiliations:** Regenerative Medicine Cluster, Advanced Medical and Dental Institute (AMDI), Universiti Sains Malaysia, Bandar Putra Bertam, 13200 Kepala Batas, Penang Malaysia

## Abstract

Airway stem/progenitor epithelial cells (AECs) are notable for their differentiation capacities in response to lung injury. Our previous finding highlighted the regenerative capacity of AECs following transplantation in repairing tracheal injury and reducing the severity of alveolar damage associated acute lung injury in a rabbit model. The goal of this study is to further investigate the potential of AECs to re-populate the tracheal epithelium and to study their stimulatory effect on inhibiting pro-inflammatory cytokines, epithelial cell migration and proliferation, and epithelial-to-mesenchymal transition (EMT) process following tracheal injury. Two *in vitro* culture assays were applied in this study; the direct co-culture assay that involved a culture of decellularised tracheal epithelium explants and AECs in a rotating tube, and indirect co-culture assay that utilized microporous membrane-well chamber system to separate the partially decellularised tracheal epithelium explants and AEC culture. The co-culture assays provided evidence of the stimulatory behaviour of AECs to enhance tracheal epithelial cell proliferation and migration during early wound repair. Factors that were secreted by AECs also markedly suppressed the production of IL-1β and IL-6 and initiated the EMT process during tracheal remodelling.

## Introduction

The respiratory airway is composed of a pool of several types of differentiated epithelial cells, such as basal, ciliated and secretory cells, that are relatively stable, in steady state, and individually have a specialised function that helps maintain the integrity of the respiratory epithelium. The respiratory epithelium is also an example of a slowly renewing tissue^1^ due to its low mitotic index, which as a results from infrequent proliferation of stem/progenitor cells in this niche. In contrast, the epithelial cell turnover rate is considerably faster in other organs such as the gut and intestine because the epithelium lining in these organs requires rapid proliferation and has an active mitotic compartment to modulate homeostasis^2^. The limited reparative capacity of the endogenous airway stem/progenitor cells becomes even lower with increasing age^3^. Lung failure due to aging can be traced to deterioration of lung stem cell population in its niche can result in impaired repair and chronic scarring^4^. Thus, the search for reparative cells that can contribute to the process of trachea repair and regeneration has become an engaging research topic, as such cells are needed for cell therapy and tissue engineering to support treatment of extensive lung injuries/disorders.

During the early stages of epithelial regeneration, the endogenous epithelial cell proliferation, migration, and differentiation are highly regulated by growth factors, cytokines, and proteases released the by airway microenvironment, neighbouring cells, and immune cells. The process of airway epithelium repair begins with damaged cells sending paracrine signals to neighbouring epithelial cells. In the trachea and bronchi region, for example, the population of basal cells that act as ‘stem cells’ receives signal and responds to injury via cell migration, proliferation, and differentiation processes^5,6^. Cell migration is one of the first mechanisms of epithelial repair. In the early repair stage, epithelial cells form a multiple layer of flattened epithelial cells^5,7^, which are associated with cytoskeleton reorganisation, membrane cell elongation, and release of adhesion proteins (cadherin, integrin, etc.) along with extracellular matrix (ECM) to facilitate the spreading and migration of the cells^6,8,9^. This phase is normally referred to as the epithelial-to-mesenchymal transition (EMT). This event is crucial and usually occurs spontaneously during wound healing or tissue remodelling^10^. The EMT involves the transition by which non-motile epithelial cells gain motility, migratory, and invasive properties to become mesenchymal stem cells (MSCs)^10,11^. The initiation of the EMT is marked by the phenotype switch from epithelial to mesenchymal cell marker such as N-cadherin^11–13^ to promote changes in epithelial cytoskeletal structure into a spindle shape morphology to acquire a more motile and mesenchymal phenotype^10,11^. Transforming growth factor-beta (TGF-β) is normally highly expressed during the EMT process in lung diseases such as idiopathic pulmonary fibrosis^14^ and asthma^15^, it also stimulates fibroblast proliferation to increase the production of ECM^16–18^. Once the epithelial barrier is re-established, the epithelial cells within the basal compartment undergo ciliogenesis or differentiate into secretory cells to re-establish pseudostratified mucociliary epithelium^5,19^.

Stem/progenitor cells of the airway have received enormous attention because they may be good candidates for cell therapy or tissue engineering. The ability to generate airway epithelial cells (AECs) from embryonic stem cells^20,21^ and induce pluripotent stem cells^22,23^ *in vitro* has provided hope that these cells may be useful in regenerative medicine approaches. Studies have suggested that airway stem/progenitor epithelial cells are notable for their self-renewal and differentiation capacities in response to lung injury. For example, studies using viral infection-induced injury^24^ and stem cell ablation-induced injury^25^ demonstrated remarkable alveolar repair involving distal airway-derived stem cell transplantation. Our previous study demonstrated a positive effect of aerosol-based AEC delivery with wide distribution of AECs into the respiratory bronchioles and lung interstitial space^26^. The delivered AECs modulated tracheal epithelium repair and regeneration, reduced inflammation, and attenuated lung injury^26^. Other studies have reported that AECs produces interleukin (IL)-10^27^ which inhibits pro-inflammatory cytokines including tumour necrosis factor (TNF)-α, IL-1β, IL-6, Macrophage inflammatory protein (MlP)-1a, and IL-8 that are produced by monocytes or macrophages^28,29^. The aim of the current study was to investigate the functional effect of AECs during tracheal repair and regeneration. Direct and indirect co-culture assays were applied in this study. We investigated the potential of AECs to re-populate the tracheal epithelium, and we assessed their effect on pro-inflammatory cytokines IL-1β and IL-6, epithelial cell migration and proliferation, and the EMT process following tracheal injury. These findings will provide insight into the role of AECs in tracheal repair and regeneration with the goal of possible application in tissue engineering and therapeutic treatment for airway injury.

## Results

### Tracheal epithelium regeneration and repair using the direct co-culture assay

**Figure 1:**
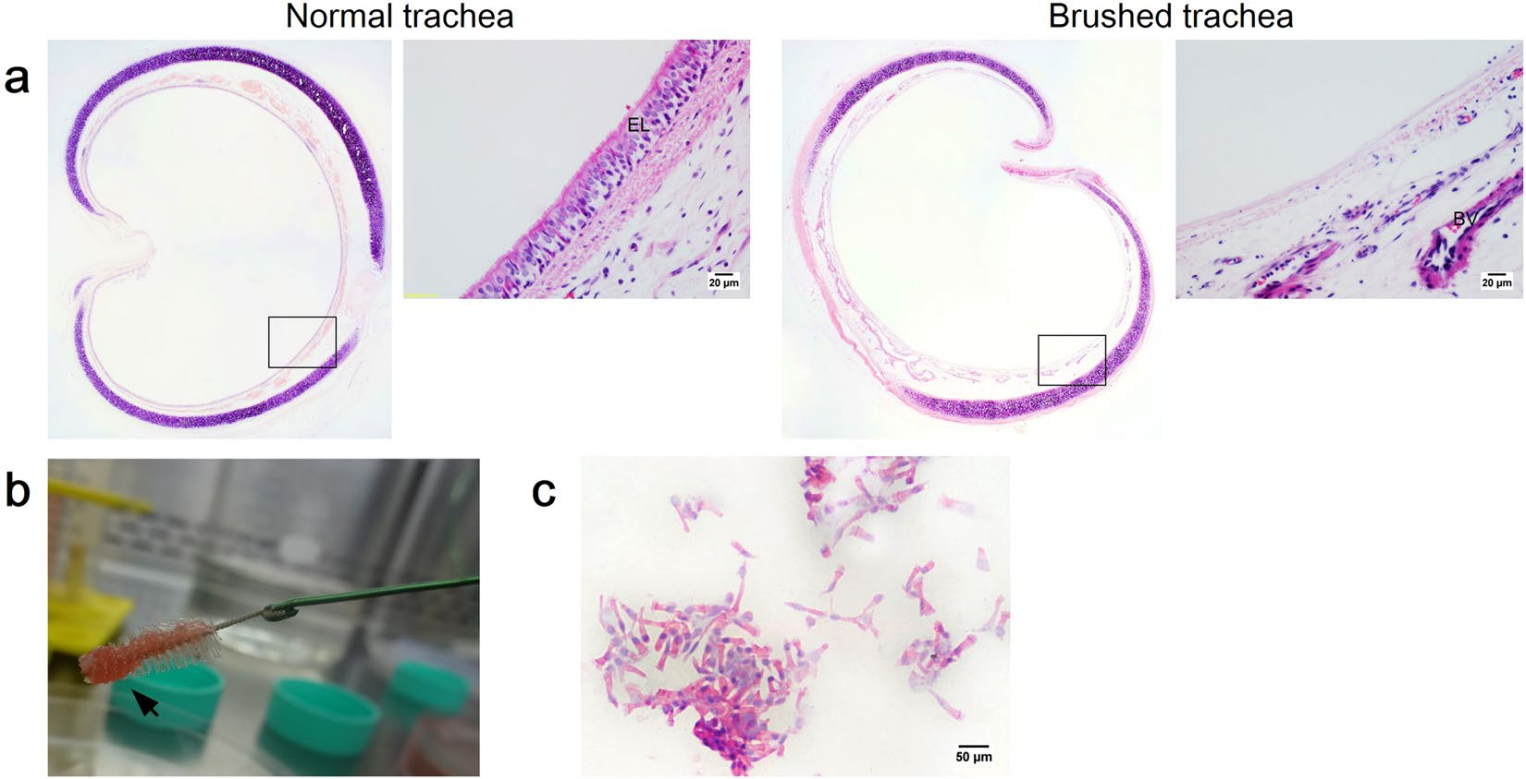
(**a**) H&E stained trachea sections from the normal and injury groups for direct and indirect co-culture assays: (left panel) Normal group with intact pseudostratified epithelium, and (right panel) injury group following brushing-induced tracheal injury. Insets show the epithelium layer at higher magnification. (**b**) Brushing technique ﻿removes tracheal epithelium layer. (**c**) H&E stained cell collection from tracheal brushing, which predominantly consists of ciliated cells (scale bars (**a**) = 20 μ﻿m, (**c**) = 50 μm).

To assess the potential of AECs to regenerate *in vitro* rabbit trachea epithelium after injury, the denuded tracheal explants were subsequently cultured with AECs. This model allowed us to examine AECs engraftment and their ability to regenerate whole tracheal epithelium. The brushing technique was performed to create “injury” and remove whole epithelium lining﻿ (Fig. 1a). The cells collected in the interdental brush bristle revealed that this technique had successfully removed epithelial cells (Fig. 1b, c).

**Figure 2:**
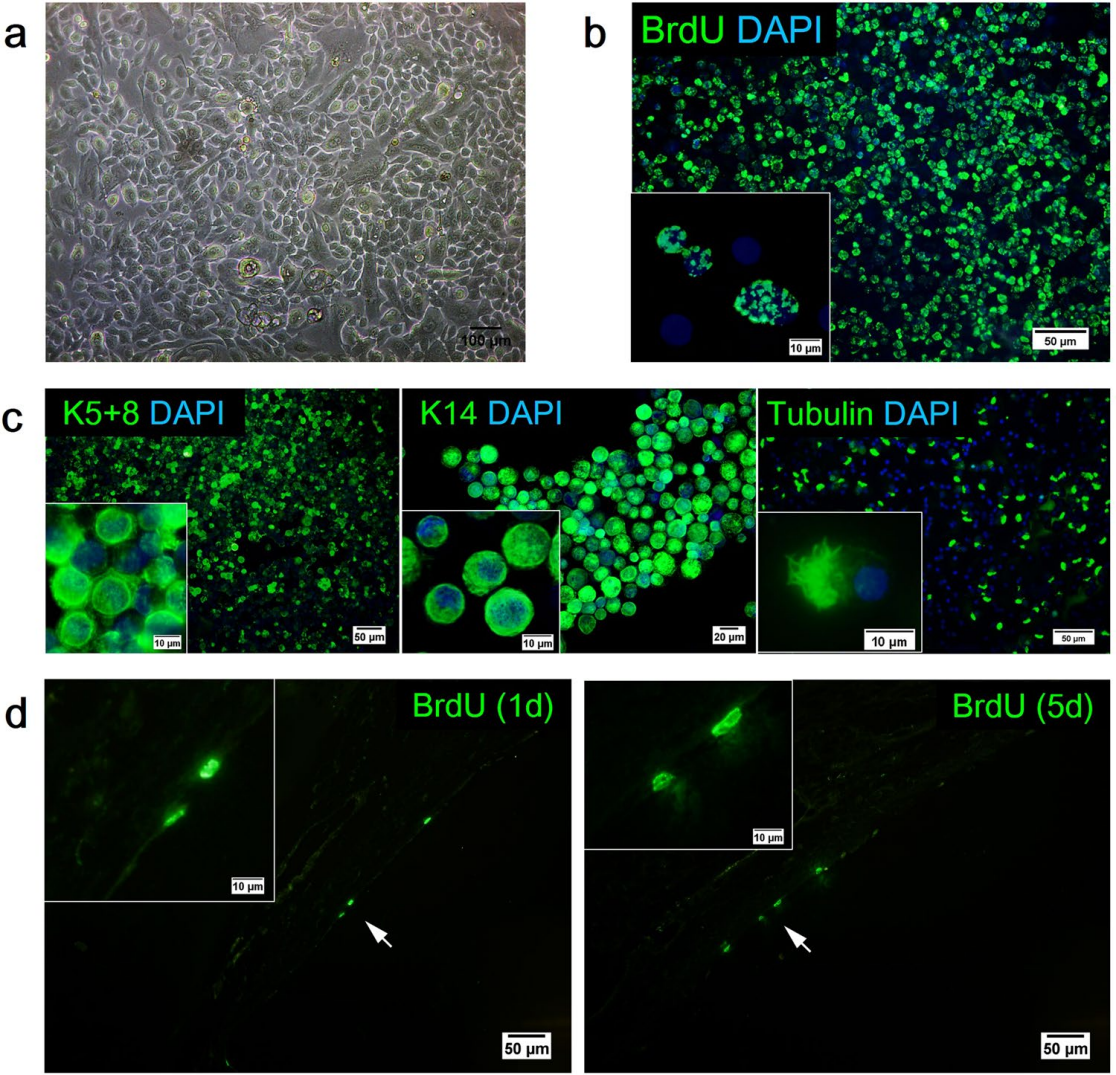
AEC morphology and characterisation. (**a**) Phase-contrast micrographs of confluent monolayer of AECs with cobblestone-like appearance. Representative images of DAPI (blue) and (**b**) BrdU (green) as well as (**c**) epithelial cell marker K5+8 (green), K14 (green), and tubulin (green) staining of AECs following isolation and culture. The detection of Br﻿dU-labelled cells on the trachea sections following direct co-culture assay. (**d**) H&E stained trachea sections of anti-BrdU staining (green) following direct c﻿o-culture assay (d1 = day 1, d5 = day 5). Arrows indicate BrdU+ cells that formed the lining of epithelium. Insets show the cell(s) at higher magnification (scale bars (**a**) = 100 μm, (**b, c, d**) = 50 μm, scale bars of insets represent 10 μm).

AECs isolated from the tracheobronchial tissue exibited epithelial cell morphologies that were identified by mosaic-like or cobblestone appearance (Fig. 2a). Tracheal epithelial cell markers such as Keratin 5 + 8 (K5+8), Keratin 14 (K14), and tubulin were apparent and expressed in the AEC culture (Fig. 2c). Green fluorescence indicated the presence of K5+8 and K14 in the cytoplasm region and the presence of tubulin in the cilia of the cells.

**Figure 3:**
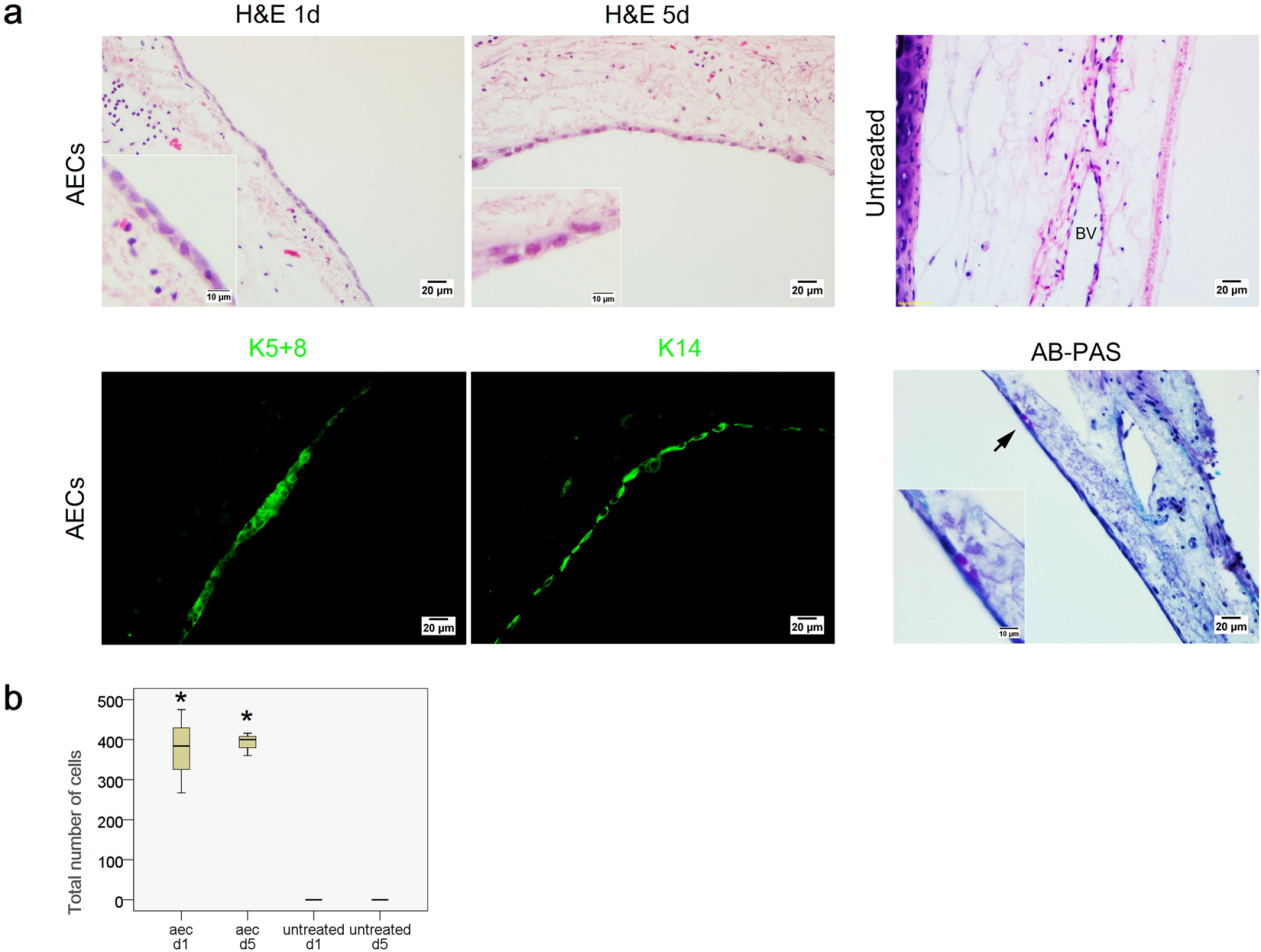
(**a**) H&E and immunofluorescence-stained trachea sections following AEC treatment in the direct co-culture assay. (upper panel) Formation of a new lining of epithelium layer in the basement membrane of the AEC-treated group at days 1 and 5. No epithelial cell detected in the base﻿ment membrane of the untreated group. (below panel) The characterization of engrafted cells following direct co-culture assay. Representative images of epithelial cell mark﻿ers, K5+8 (green) and K14 (green) and AB-PAS stained tracheal segment. Purple staining indicates mucus producing cells. Insets show the epithelium layer at higher magnification (scale bars = 20 μm, scale bars of insets represents 10 μm). (**b**) Boxplot illustrates the quantification of epithelial c﻿ells that engrafted in the basement membrane of the trachea following AEC delivery at days 1 and 5. Upper and lower boxplot margins represent the interquartile range and the middle bar indicates the median. The whiskers define the range of values (d1 = day 1, d5 = day 5, BV = blood vessels). Data are represented as mean ± standard deviation; n = 3 trach﻿eal tissue for each group (untreated vs. aec-tre﻿ated, *P < 0.05).

Figure 3a showed a layer/multilayer of flattened epithelial cells lined the basement membrane after initial seeding of the AECs direct co-culture. At day 5, these flattened cells remained attached in the trachea. The total number of cells counted in the whole trachea at day 5 was relatively reflected to the number at day 1 post AECs direct co-culture (AEC d1 375.33 ± 104.27 vs. AEC d5 392 ± 28.84) (Fig. 3b). In contrast, the trachea in the untreated group remained denuded when culture was prolonged up to 5 days.

The flattened cells were further characterised using epithelial markers (K5+8, K14) and alcian blue-periodic acid-Schiff (AB-PAS) staining (Fig. 3a, more evidence for K5+8^+^ and K14^+^ cells can be found in supplementary Figure 1). The engrafted cells were positive for both K5+8 and K14, demonstrating that nearly all flattened cells expressed basal and ciliated epithelial cell markers. A few of these epithelial cells also stained with AB-PAS, indicating the development of secretory cells. Before co-culture, the cells were labelled by *in vitro* bromodeoxyuridine (BrdU) incubation (81.16 ± 1.82% of labelling efficiency) (Fig. 2b). Anti-BrdU staining at day 1 and 5 indicated that, the BrdU^+^ flattened epithelial cells originated from the engrafted AECs (Fig. 2d, more evidence for BrdU^+^ epithelial cells can be found in supplementary Figure 2).

### Tracheal epithelium regeneration and repair using the indirect co-culture assay

**Figure 4:**
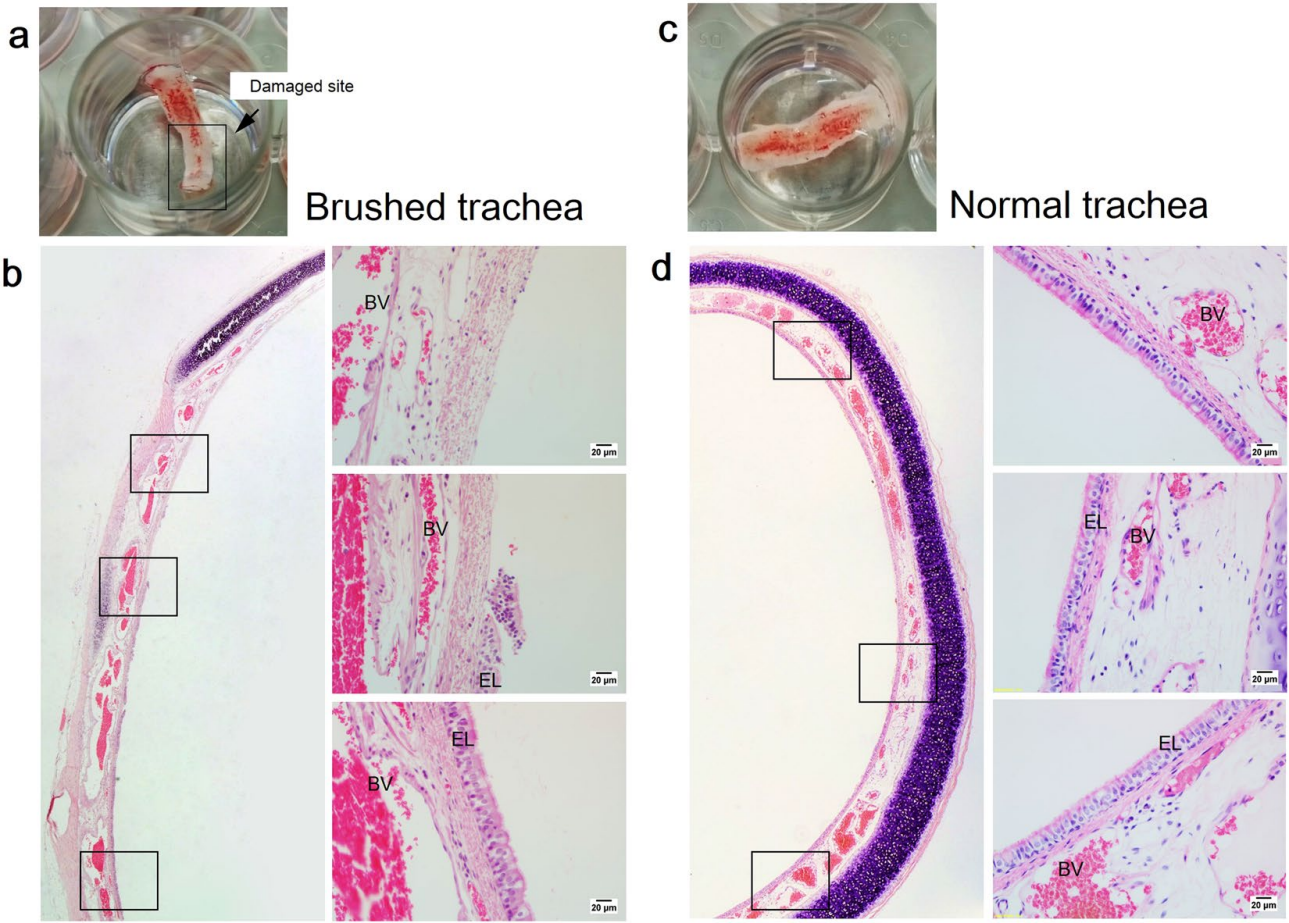
(**a**) Gross examination of the tracheal tissue explant after exposed to brushing-induced injury. (**b**) H&E stained trachea sections from the injury group for indirect co-culture assays The partially brushing technique divided the trachea into two regions, denuded region and undamaged region. (**c**) Gross examination of the normal tracheal tissue explant. (**d**) H&E stained trachea sections from the normal group with intact pseudostratified epithelium. Insets show the epithelium layer at higher magnification. (EL = epithelium layer; BV = blood vessels) (sscale bars of insets represent 20 μm).

In this model system, the injury was initiated by cutting the connective tissue that holds the tracheal ring, thus leaving the luminal trachea exposed to air (Fig. 4a). Histological examination of the tracheal explants showed that the brushing procedure removed part of the epithelium lining but left the other part undisturbed (Fig. 4b). In contrast, the uninjured trachea explant maintained its formation of pseudostratified epithelium with evidence of intact epithelial cells lining the basement membrane (Fig. 4d). Figure 4b demonstrated that this type of injury divided the trachea into two different regions, damaged and undamaged, which consisted of pseudostratified epithelium.

**Figure 5:**
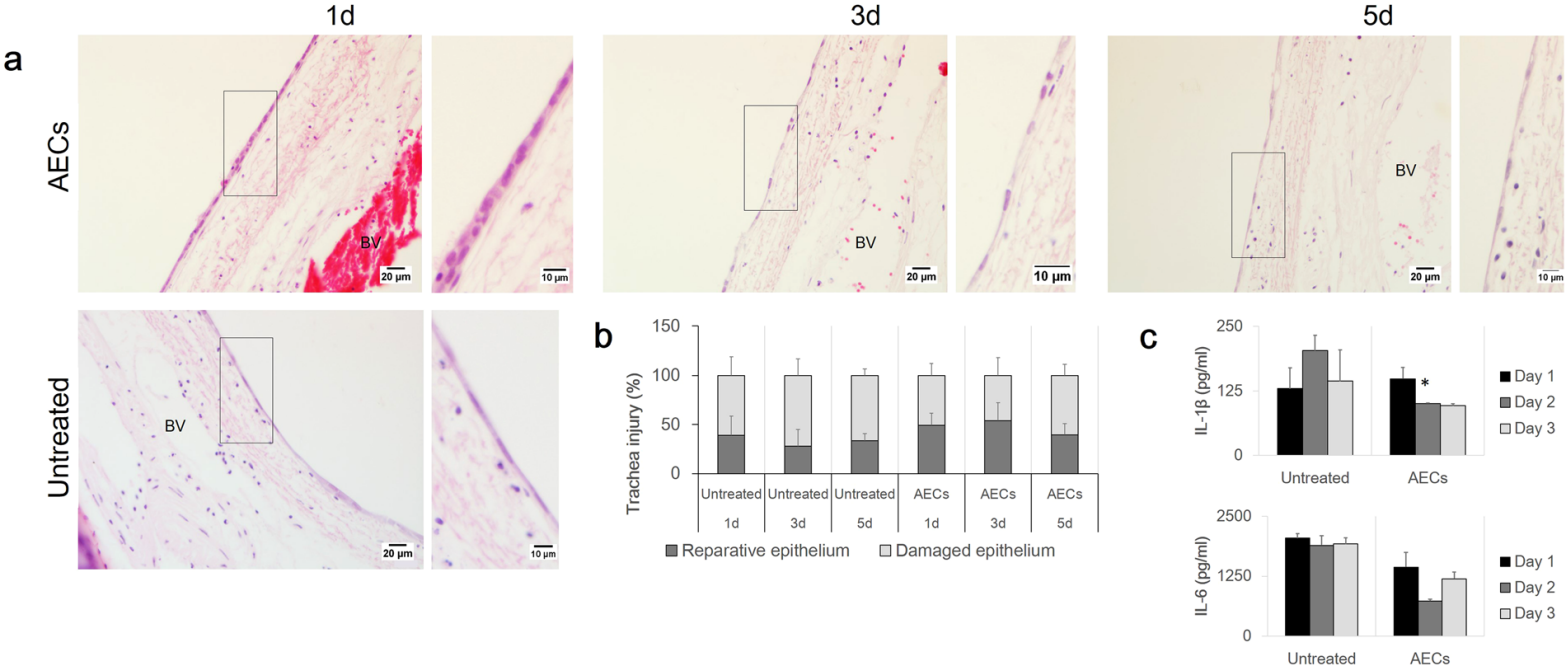
(**a**) H&E stained trachea sections following cell treatment in indirect co-culture assay. De-differentiation of a pseudostratified epithelium into flattened epithelial cells was noted in both AEC-treated and untreated group. Inset shows the epithelium layer at higher magnification.(**b**) The graph illustrates the percentage of damaged and reparative epithelium region following indirect co-culture assay. (**c**) The graph illustrates the level of IL-1β and IL-6 inflammatory cytokines in response to AEC treatment. Data are represented as mean ± ﻿standard deviation; n = 3 tracheal tissue for each﻿ group (untreated vs. aec-treated, *P > 0.05) (d1 = day 1, d3 = day 3, d5 = day 5) (scale bars = 20 μm, scale bars of insets represent 10 μm).

**Figure 6:**
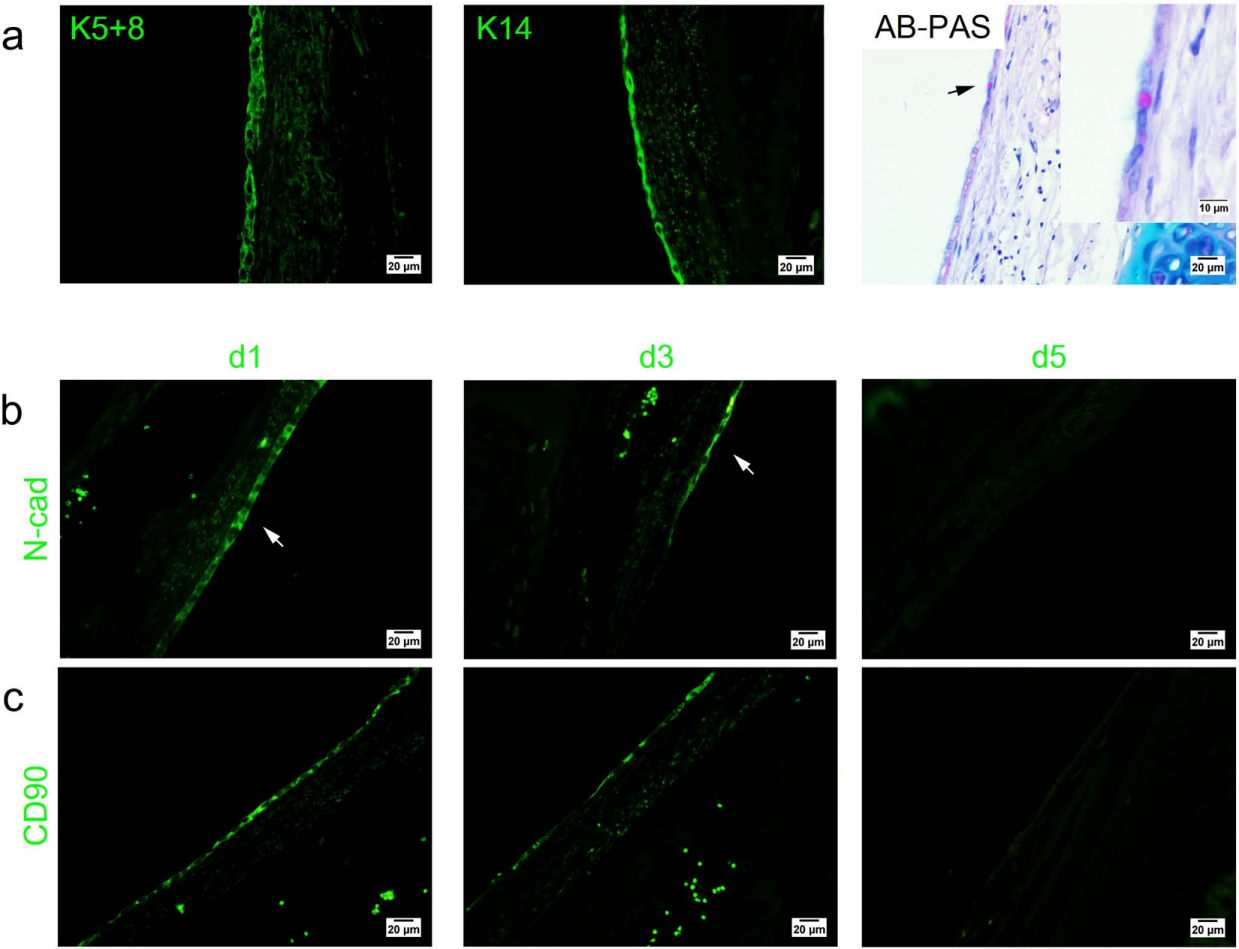
(**a**) The characterization of engrafted cells following indirect co-culture assay. Representative images of epithelial cell markers, K5+8 (green), K14 (green), and AB-PAS stained tracheal segment. Magenta staining indicates mucus producing cells. Inset shows the cell at higher magnification. (**b**) Representative images of EMT markers, N-cadherin (green) and CD90 (green) (d1 = day 1, d3 = day 3, d5 = day 5) (scale bars = 20 μm, scale bars of inset represents 10 μm).

Figure 5a illustrates that tracheal repair occurred very rapidly. Within 1 day, the undamaged region of the trachea had de-differentiated into squamous epithelial cells to prepare for migration and wound repair. In the AEC-treated group, tracheal basement membrane surfaces on day 1 were uniformly lined by a mono- to bilayer formation of cuboidal epithelium with rounded nuclei and adjacent cells close to each other (Fig. 5a, day 1), whereas the epithelial cells of the untreated group appeared to form just one layer with elongated nuclei and cytoplasm and with adjacent cells distant from each other (Fig. 5a, untreated). The epithelial cell shape on days 3 and 5 following AEC treatment also appeared to be more flattened, with mesenchymal-cell-like morphology, similar to the appearance of the untreated cells Fig. 5a, days 3 and 5). The percentage of the epithelial lining count indicated that secretory factors in the AEC culture medium had stimulated migratory behaviour of these squamous epithelial cells during wound repair as compared to the untreated group (Fig. 5b) (AEC d1 49.38 ± 12.32% vs untreated d1 39.25 ± 19.23%). Epithelial cell migration to the denuded region is proposed as an important element of wound repair and regeneration in tracheal injury. Epithelial reparative activity peaked at day 3 and declined at day 5, and this pattern was similar to that observed in the untreated group (Fig. 5b). However the percentage of reparative epithelium was markedly higher in the AEC-treated group compared with the untreated group (AEC d1 49.38 ± 12.32%, AEC d3 52.88 ± 18.21%, AEC d5 39.42 ± 11.41% vs untreated d1 39.25 ± 19.23%, untreated d3 28.07 ± 16.81%, untreated d5 33.75 ± 6.88%). Figure 6a suggested that these epithelial cells exhibited the basal and ciliated epithelial cell-specific markers K5+8 and K14 and were positive for AB-PAS staining (more evidence for K5+8^+^ and K14^+^ cells can be found in supplementary Figure 3).

At day 1, both N-cadherin (Fig. 6b) and CD90 (Fig. 6c) expression were highly expressed in tracheal explants of the AEC-treated group, indicating the initiation of EMT process in response to the stimulatory factors released by AECs (more evidence for N-cadherin^+^ and CD90^+^ cells can be found in supplementary Figure 4). N-cadherin expression was retained until day 3 and was absent at day 5 in the AEC treatment group. A similar pattern of expression was also observed for CD90 staining, the expression of both mesenchymal markers was noted at days 1 and 3, and disappeared at day 5 following AEC treatment. Together, these findings suggested that metabolic components released by AEC potentially induced N-cadherin and CD90 expression thus indicating the initiation of the EMT process during early repair of tracheal epithelium.

### IL-6 and IL-1β level in AEC-conditioned medium

Pro-inflammatory cytokines IL-6 and IL-1β were two of the major cytokines present during airway injury. AEC treatment in our *in vitro* model system reduced the level of both IL-1β and IL-6 (Fig. 5c). The level of IL-1β cytokine level was attenuated at day 2 and slightly reduced at day 3 compared with the level in the untreated group (d2 203.64 ± 29.24 vs. 101.24 ± 0.8, d3 144.37 ± 60.51 vs. 96.74 ± 3.78 pg/ml, *P < 0.05). A similar pattern was observed for the IL-6 level. Addition of AEC culture at the top of the tracheal explant resulted in inhibition of IL-6 level in the AEC-treated conditioned medium (d1 2045.94 ± 95.74 vs. 1441.97 ± 307.57, d2 1894.84 ± 204.67 vs. 735.95 ± 37.97, d3 1924.06 ± 133.13 vs. 1196.92 ± 138.34 pg/ml). Inhibition of IL-6 and IL-1β by AEC was associated with the increased proliferation and migratory activities of tracheal epithelial cells after injury and also with the EMT process (Figs 5c and 6b,c).

## Discussion

The direct co-culture assa﻿y in this study was designed to investigate the ability of AECs to reconstruct a new tracheal epithelium layer. Within 24 hours, a compact layer of flattened epithelial cells that originated from AECs uniformly lined the basement membrane of the trachea. After 5 days of incubation, the engrafted AECs retained the expression of basal and ciliated epithelial cell markers such as K5+8, K14, and positive for AB-PAS staining. However, the appearance of the epithelial cells indicated a poorly differentiated cells, considering that within five days, the cells were still in their ‘reparative’ state. We hypothesise that the AEC on the tracheal construct in the direct co-culture assay may form cilia and differentiate over time. The indirect co-culture assay used in this study resulted in a decrease of IL-1β and IL-6 pro-inflammatory cytokines, an increase in early epithelial cell proliferation and migration, and also initiation of the EMT process during early tracheal regeneration and repair following the addition of the AEC culture at the top of the tracheal defect. These results indicated that AEC might release secretory factors that that stimulated early tracheal epithelial repair and regeneration.

AEC that portrayed in this study comprised of different epithelial cell types that majorly expressed K5+8 (ciliated and basal cells) and K14+ (basal cells). The interplay of intracellular signalling between these epithelial cells is crucial in order to initiate differentiation during airway regeneration and repair. Basal cells are marked by the expression of K5 and K14. These cells represent a multipotent progenitor cell type for renewal of the injured tracheal epithelium epithelium^30,31^. K14-expressing basal cells after naphthalene injury were capable of both self-renewal and giving rise to ciliated and secretory cells *in vivo* ^30^. Furthermore, p63/K5-cells that arose from secretory cells after basal cells ablation-induced injury acquired the potential plasticity of basal cells and had the ability to repopulate the denuded airway^32^. In conditions which epithelial cells such as secretory and ciliated cells were completely ablated by SO_2_ exposure, suprabasal cells or early progenitor cells that were derived from basal cells started to express K8, which is a marker for ciliated cells. This occurrence indicated that these basal cells had the ability to generate ciliated and secretory lineages^33^. Similar events were observed in our study, as the lining of flattened epithelial cells in the direct co-culture system not only expressed K5+8 but also K14 and were positive for AB-PAS staining (Fig. 3a, Supplementary Figure 1). The expression of K14 indicated that these cells were activated and under steady state conditions to differentiate. In addition, this lining of cells were originated from the seeded AECs that attached to the basement membrane when placed in a direct contact with the damaged tracheal explant. However, since this study provided only 5 days of observation, we proposed that over a longer period of time these cells would have completely repopulated by basal epithelial cells that followed by differentiation into mucous and ciliated epithelial cell, thus formed pseudostratified epithelium layer. This assumption was based on a few studies in a tracheal engineered model that suggested 4^34,35^ to 5^36^ weeks of incubation time to completely reconstruct mucociliary pseudostratified epithelium layer.

In the direct co-culture study, epithelial cell count suggested that the proliferation of AECs after engraftment was occurring at a very slow rate (Fig. 3b). The lack of AEC proliferation and differentiation to form a pseudostratified epithelium in our experiment might be due to the absence of interaction between epithelial cells and stromal cells. *In vivo*, cell signalling between epithelial and stromal cells plays an important role in fate induction processes during airway epithelium repair^37^. A tissue niche is usually composed of cellular components, which may include epithelial, endothelial, stromal (mesenchymal/fibroblasts), and/or hematopoietic cells. These cells interact with the endogenous stem cells through signalling components, which may be paracrine or autocrine signals. For example, the interaction between alveolar type II (ATII) cells with lung fibroblasts in an *in vitro* culture resulted an emergence of Wnt signal^38^, a cell signalling that is not only essential and marked during early lung development^39–41^, but also has been implicated in the modulation of cell fate decisions and differentiation of lung cell types^38,42^. Stromal cells are also critical regulators of alveolar homeostasis, and addition of lung stromal cells is required to form alveolar spheres *in vitro* ^43^. Thus, it is understandable that the airway repair process is influenced by a combination of signalling from cells such as epithelial and stromal cells.

In addition to cell-to-cell signalling, ECM acts as a matrix that sustain the microenvironment of a niche such as endogenous stem cell quiescence and self-renewal *in vivo* ^44^. Substances such as collagen, laminin, and fibronectin also guide the cell fate decisions, which include regulating cell proliferation, self-renewal or differentiation, migration, quiescence, and cell death^45,46^. We believe that these components were absent in this direct co-culture study, which led to the lack of AEC proliferation and differentiation from flattened epithelial to pseudostratified epithelium after 5 days of culture. Flattened cells indicate poorly differentiated cells that are still in the early phase of regeneration^7,47^. Therefore, we conclude that a longer incubation time with a suitable ECM substrate and/or the addition of stromal cells should be used to stimulate the proliferation of flattened epithelial cells and lead to a fully developed pseudostratified respiratory epithelium.

According to several studies, AECs secrete factors such as IL-10^27^, vascular endothelial growth factor α^48^, and mucin proteins^49^. The addition of AEC culture in the indirect co-culture system was accompanied by dynamic changes in the levels of IL-6 and IL-1β in the conditioned medium. Levels of both IL-1β and IL-6 were reduced in the AEC-treated group compared to the untreated trachea group following injury (Fig. 5c). Our histology assessment demonstrated that the percentage of reparative epithelium was higher in the AEC-treated group compared to the untreated group, which reflected an ongoing regeneration of epithelium following injury (Fig. 5b). Thus, we speculated that AECs released anti-inflammatory factors that suppressed pro-inflammatory cytokines (i.e., IL-10 vs. IL-1β and IL-6), thereby protecting and stimulating the migration and proliferation activities of the remaining epithelium to reconstruct the denuded region of the tracheal explant. Fiorentino *et al*. demonstrated that constitutive IL-10 secretion inhibited the production of a number of pro-inflammatory cytokines, including TNF-α, IL-1, IL-1α, and IL-6^28^. Thus, IL-10 was released by AECs likely played a role in reducing the levels of inflammatory cytokines IL-1β and IL-6. However, the stimulatory mechanism of epithelial proliferation, migration and attenuation of inflammatory cytokines during tracheal regeneration and repair needs to be clarified further.

In the indirect co-culture study, the remaining epithelial cells in the AEC-treated group underwent mesenchymal phenotypic changes, which were consistent with the expression of the EMT marker, N-cadherin, and the mesenchymal marker, CD90. The untreated group, in contrast, was negative for both N-cadherin and CD90 markers. Additionally, at days 3 and 5, differentiation of the epithelial cells had resulted in flattened cells with a more mesenchymal morphology (Fig. 6b,c, Supplementary Figure 4). These cells, however, still exhibited markers for epithelial cells (K5+8 and K14) but had a mesenchymal-like cell morphology, a spindle shape, and an elongated cytoplasm. Similar to our results, studies by Nishioka *et al*. and Sohal *et al*. also reported that epithelial protein such as E-cadherin^13^ and epithelial growth factor receptor^50^ were expressed and maintained in addition to an increase of fibroblast/mesenchymal markers such as N-cadherin, Vimentin^13^, matrix metalloproteinase-9, and fibroblast specific-protein-1^50^ during EMT process.

In this study, it is currently unclear which factor in the AEC-conditioned medium was responsible for inducing the mesenchymal-like phenotype in the remaining epithelial cells. This action may be mediated by TGF-β. TGF-β is a cytokine/growth factor that plays a critical role in the modulation of tissue repair as a wound healing promoting factor^51^ and in the progression of fibrosis; it also induces the EMT in lung diseases such as idiopathic pulmonary fibrosis^14^ and asthma^15^. According a several studies, TGF-β is produced by a number of cells, including macrophages, epithelial cells, fibroblasts, and eosinophils^52,53^ and plays a critical role in the modulation in tissue repair as wound-healing promoting factors. In this case, further studies are necessary to include the possibility of AECs in releasing TGF-β, which was associated with initiation of the EMT process.

In summary, the current findings indicated that the direct and indirect *in vitro* model systems represented an appropriate model for investigating early tracheal regeneration and repair. This report provides an evidence to support the data from our previous *in vivo* study^26^ on AEC transplantation in the setting of an acute lung injury model. AEC direct co-culture resulted in attachment and re-population of the de-epithelised trachea with flattened epithelial cells. However, a longer period of incubation time with suitable ECM compounds, and/or the addition of stromal cells are needed to stimulate the proliferation of flattened epithelial cells which would lead to a fully developed pseudostratified respiratory epithelium. The robust cell proliferation, migration, and marked expression of K5+8, K14, CD90, and N-cadherin following incubation with AEC culture appeared to be associated with the EMT process during early tracheal remodelling. These occurrences also suggested that factors secreted by AECs may serve as therapeutic agents to induce repair of respiratory epithelium following injury.

## Methods

### Animals

A total of seven adult female New Zealand white rabbits with average weight of 2.5 ± 0.29 kg were used in this experiment. The whole trachea tissue from the three rabbits were harvested and cut into tracheal segments. Meanwhile, AECs were derived from the tracheobronchial tissue of the other four female rabbits. All rabbits in this experimental study were purchased from Animal Research and Service Centre, Universiti Sains Malaysia (USM) and were housed at the Research Animal Facility of the Advanced Medical and Dental Institute, USM. All animal procedures were approved and performed according to the ethical standards of the Animal Ethics Committee of the Universiti Sains Malaysia (USM/Animal Ethics Approval/2014/(91) (537).

### Isolation of tracheal tissue

The rabbits were euthanized by intravenous injection of pentobarbital (200 mg/ml). Each trachea was harvested using a standard surgical procedure. The trachea was excised between the larynx and the bifurcation into the main stem bronchi. The trachea was removed and transferred into ice-cold 1x phosphate-buffered saline (PBS). AECs were isolated from four of the collected tracheas, whereas each of the other three whole tracheas was cut into 10 pieces, yielding a total of 30 pieces (approximately 1 cm long) for *in vitro* trachea decellularisation. Unless stated otherwise, all reagents were purchased from Gibco-Thermo Fischer Scientific (Massachusetts, US).

### Isolation and characterisation of AECs

AECs were isolated from the tracheobronchial of rabbits based on a previously described protocol^54^. Briefly, the excised trachea was collected and transferred into dissociation medium containing Ca^2+^ and Mg^2+^ free Minimum Essential Medium, pronase from *Streptomyces griseus* (Roche, Basel, Switzerland), DNase I from bovine pancreas (Roche, Basel, Switzerland), and the antibiotic-antimycotic solution. The trachea in dissociation medium was then incubated using a MACSmix™ tube rotator (Miltenyi Biotec, Bergisch Gladbach, Germany) (setting: permanent run at a speed of 12 RPM) at 4 °C overnight. After 24 hours of incubation, the epithelial part of the trachea piece was separated from the cartilage by gently scraping with a scalpel. A single cell suspension was obtained by filtering both the tissue suspension and the scraped epithelial cell through 70 µm cell strainer. Cell culture flask, petri dish, and well-plate were coated with human placenta collagen type IV (Sigma-Aldrich, Missouri, US), and air-dried inside the biosafety cabinet for overnight. The cells were then cultured in serum-free Bronchial Epithelial Growth Medium (BEGM™) (Lonza, Basel, Switzerland) (BEGM™ consisted of Bronchial Epithelial Basal Medium, bovine pituitary endocrine, hydrocortisone, human epidermal growth factor, epinephrine, transferrin, insulin, retinoic acid, and triiodothyronine) and cultured at 37 °C in a 5% CO_2_ incubator. To obtain AEC culture with less fibroblast contamination, we removed some of the fibroblast cells when sub-plating the culture for a new passage. Since fibroblasts cells attach more quickly compared to epithelial cells, fibroblast cells were allowed to adhere for 10 minutes, the epithelial cells were then removed and re-plated into a new culture flask. AEC culture from passages 3 to 5 were used in this experiment. The characterisation of AECs was performed using antibody detection of K5+8 for basal and ciliated cells (Cat. no. AB9005, dilution 1:500), K14 for basal cells (Cat. no. AB77684, dilution 1:200) and tubulin for ciliated cells (Cat. no. AB56676, dilution 1:200) (Abcam, Cambridge, UK).

### Direct co-culture assay

**Figure 7:**
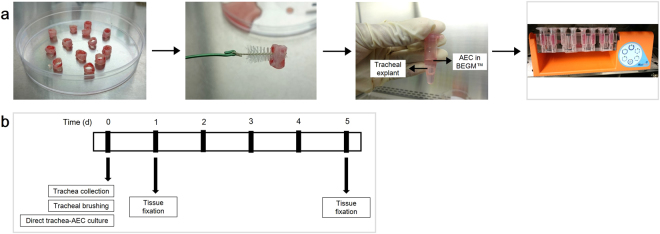
(**a**) Experimental design of direct co-culture assay. One whole trachea was cut into ten pieces of trachea segments. Trachea was de-epithelized using an interdental brush. The tracheas with/without AECs were placed in a microcentrifuge tube containing BEGM™. The tube was then rotated in MACSmix™ tube rotator continuously. (**b**) Timeline of the experimental protocol for direct co-culture assay.

The direct co-culture assay was performed to assess the *in vitro* regeneration of the rabbit trachea epithelium after injury. The procedure consisted of two preparations, tracheal explants with de-epithelised tracheal epithelium layer and BrdU-labelled AEC culture. At ~60% confluence, AECs were labelled by overnight incubation with 10 µM BrdU solution (Roche, Basel, Switzerland). An immunocytochemistry assay using anti-BrdU (Abcam, Cambridge, UK) was performed to detect BrdU incorporation into the nucleus. Figure 7a showed the experimental procedure of the direct co-culture assay. The epithelium of the tracheal segments were de-epithelised using an Oral-B tapered interdental brush (Procter & Gamble Co., Cincinnati, Ohio). Then, the trachea was washed with sterile 1x PBS. The BrdU-labelled AECs were harvested and counted using a haemocytometer. The trachea and AECs with a total number of 10^6^ cells were placed in a microcentrifuge tube containing BEGM™. For the control, the damaged trachea explant was cultured with BEGM™ without AECs. The tube was then rotated in a MACSmix™ tube rotator continuously (setting: permanent run at a speed of 12 RPM) at 37 °C in a 5% CO_2_ incubator. Following incubation, the trachea was collected at days 1 and 5, and fixed in 10% neutral buffered formalin. Figure 7b showed a schematic diagram of the experimental protocol.

### Indirect co-culture assay

**Figure 8:**
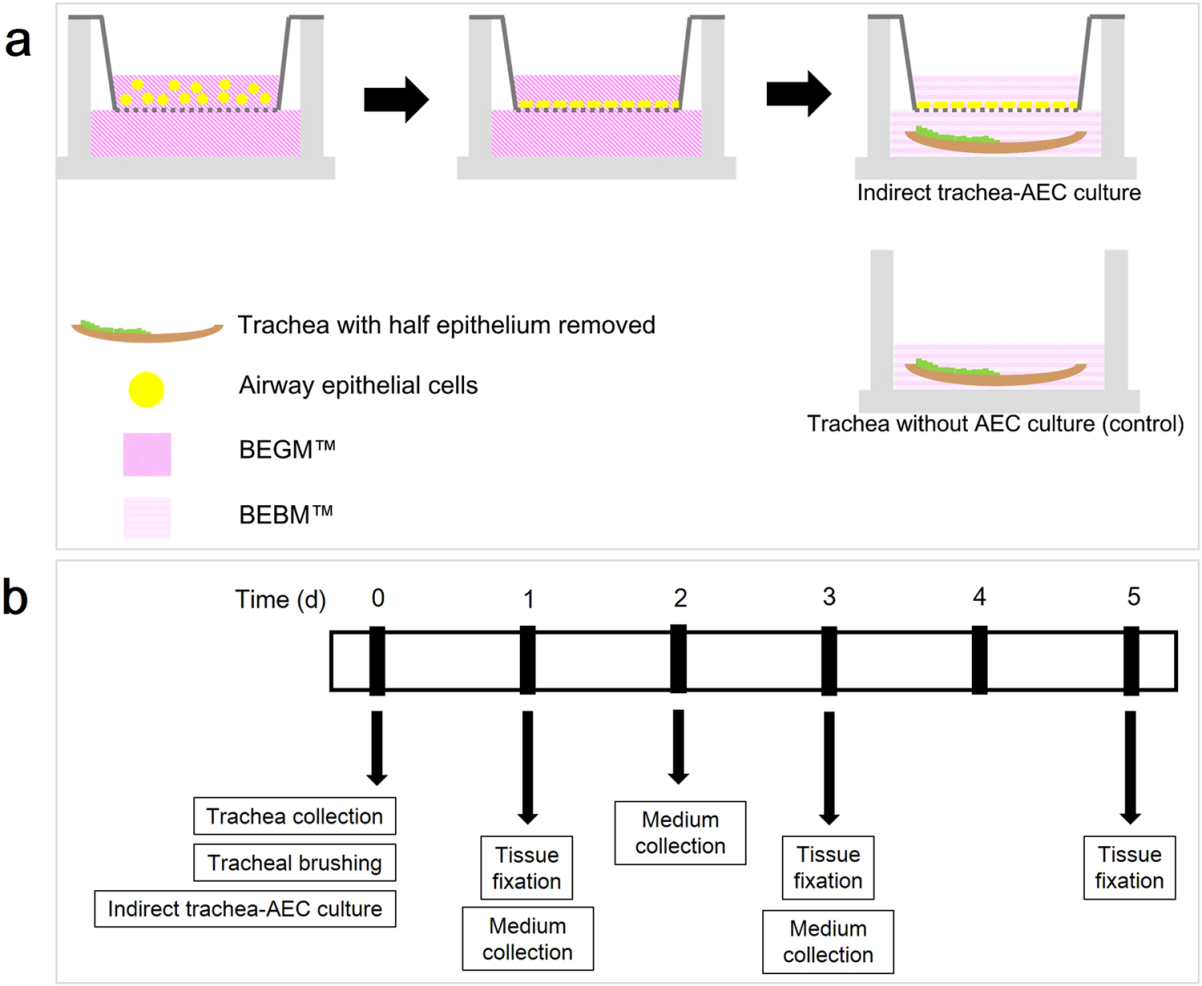
(**a**) Experimental design of indirect co-culture assay. A schematic summary of the indirect co-culture assay. A monolayer of the AEC was grown on a microporous membrane in a 24-well chamber system in BEGM™. Both tracheal explant with partially removed epithelium and starved AEC monolayer were cultured together for 1, 3, and 5 days. (**b**) Timeline of the experimental protocol for indirect co-culture assay of AEC and trachea with partially removed epithelium.

The indirect co-culture assay was performed to investigate the effect of AECs’ secreted factors on tracheal epithelial cell proliferation, migration, and initiation of EMT process in the partially damaged trachea. The procedure consisted of two preparations, tracheal explants with partially damaged tracheal epithelium layer and starved-AEC culture. Figure 8a showed the experimental design of the indirect co-culture assay. A monolayer of AECs was grown on ThinCert™, a polyethylene terephthalate microporous membrane (0.4 μm pore size in diameter) using a 24-well chamber system (Greiner Bio-One, Kremsmünster, Austria). BEGM™ was added to the lower chamber and medium was changed every 2 days. Tracheal segments were cut open and with the epithelium layer facing up, part of the trachea lining was scraped using an interdental brush, whereas the other part was left undisturbed. The trachea was then washed with sterile 1x PBS. After the AEC culture reached ~90% confluence, the cells were washed twice with PBS and then starved for 2 hours in the growth factors-free medium (BEBM™; Bronchiole Epithelial Basal Medium). Following cell starvation, the tracheas with partially removed epithelium were placed at the bottom of the chamber. Next, 600 µl of BEBM were added to the bottom of the chamber to submerge the trachea while exposing the AEC culture to air. The 24-well plate was then placed in a 5% CO_2_ incubator at 37 °C. Trachea and medium were collected at day 1, 3, and 5 of incubation. The trachea segments were then fixed in 10% neutral buffered formalin, whereas the medium was stored at −80 °C. Figure 8b shows a schematic diagram of the experimental protocol.

### Histopathological assessment of trachea following direct and indirect co-culture assays

Each trachea was embedded in paraffin and sectioned at a 3 µm thickness. H&E and AB-PAS staining were performed to observe the morphology of the trachea and to characterise the secretory cells. All staining reagents were purchased from Sigma-Aldrich (Missouri, US). All cell nuclei on the basement membrane of each direct co-cultured trachea were counted to quantitatively evaluate the re-epithelisation of the trachea following AEC treatment. In each indirect co-cultured trachea, re-epithelisation was assessed by measuring the epithelium length on the basement membrane at 100x magnification based on the following criteria: damaged and reparative. Damaged regions indicated a denuded basement membrane without epithelial cells, whereas reparative regions directed to squamous and flattened epithelial cells. This measurement was used to obtain the percentage of both damaged and reparative regions in order to evaluate the effect of the treatments on tracheal epithelium regeneration and repair. The characterisation of engrafted AECs in direct co-culture assay was observed using immunofluorescence staining of BrdU (Cat. no. AB8152, dilution 1:500), K5+8, K14, and tubulin. The epithelial cells in the indirect co-culture trachea were characterised using K5+8, K14, and tubulin. The initiation of the EMT was asse﻿ssed using two mesenchymal markers, N-cadherin﻿ (Cat. no. AB66025, dilution 1:500) and CD-90﻿ (Cat. no. AB225, dilution 1:500) (Abcam, Cambridge, UK).

### Measurement of IL-6 and IL-1β cytokine levels

The levels of IL-6 and IL-1β in growth medium exposed to AEC culture for 1, 2, and 3 days were analysed by enzyme-linked immunosorbent assay (ELISA) according to the manufacturer’s instructions. IL-6 ELISA kit was purchased from RayBiotech (Georgia, US), while IL-1β ELISA kit was purchased from R&D System (Minneapolis, US). The medium collected was proportioned into 200 µl aliquots and stored at −80 °C. 100 µl of standards and samples (3000 pg/ml) were used for the assay procedure.

### Statistical analysis

Statistical analysis was performed using SPSS ver. 22 (SPSS Inc., Illinois, US). The distribution of all data was tested for normality using Shapiro-Wilk tests. A mean from each group was compared using one-way analysis of variance with Bonferroni’s multiple comparison tests. Differences were considered to be statistically significant at *P* < 0.05. Data are represented as mean ± standard deviation; n = 3 tracheal tissue for each group.

## Electronic supplementary material

Supplementary Information

## References

[1] Stripp BR, Reynolds SD (2008). Maintenance and repair of the bronchiolar epithelium. Proc Am Thorac Soc..

[2] Weiss DJ (2015). An official American Thoracic Society workshop report: stem cells and cell therapies in lung biology and diseases. Ann Am Thorac Soc..

[3] Wetsel RA, Wang D, Calame DG (2011). Therapeutic potential of lung epithelial progenitor cells derived from embryonic and induced pluripotent stem cells. Annu Rev Med..

[4] Navarro S, Driscoll B (2017). Regeneration of the Aging Lung: A Mini-Review. Gerontology..

[5] Yahaya B (2012). Understanding cellular mechanisms underlying airway epithelial repair: selecting the most appropriate animal models. Sci World J..

[6] Girault A, Brochiero E (2014). Evidence of K+ channel function in epithelial cell migration, proliferation, and repair. Am J Physiol Cell Physiol..

[7] Dupuit F (2000). Differentiated and functional human airway epithelium regeneration in tracheal xenografts. Am J Physiol Lung Cell Mol Physiol..

[8] Su Y, Yang J, Besner GE (2013). HB-EGF promotes intestinal restitution by affecting integrin-extracellular matrix interactions and intercellular adhesions. Growth Factors..

[9] Crosby LM, Waters CM (2010). Epithelial repair mechanisms in the lung. Am J Physiol Lung Cell Mol Physiol..

[10] Zeisberg M, Neilson EG (2009). Biomarkers for epithelial-mesenchymal transitions. J Clin Invest..

[11] Lamouille S, Xu J, Derynck R (2014). Molecular mechanisms of epithelial-mesenchymal transition. Nat Rev Mol Cell Biol..

[12] Huang RY, Guilford P, Thiery JP (2012). Early events in cell adhesion and polarity during epithelial-mesenchymal transition. J Cell Sci..

[13] Nishioka M (2015). Fibroblast-epithelial cell interactions drive epithelial-mesenchymal transition differently in cells from normal and COPD patients. Respir Res..

[14] Willis BC, Borok Z (2007). TGF-beta-induced EMT: mechanisms and implications for fibrotic lung disease. Am J Physiol Lung Cell Mol Physiol..

[15] Hackett TL (2009). Induction of epithelial-mesenchymal transition in primary airway epithelial cells from patients with asthma by transforming growth factor-beta1. Am J Respir Crit Care Med..

[16] Brand OJ (2015). Transforming Growth Factor-beta and Interleukin-1beta Signaling Pathways Converge on the Chemokine CCL20 Promoter. J Biol Chem..

[17] Kitamura H (2011). Mouse and human lung fibroblasts regulate dendritic cell trafficking, airway inflammation, and fibrosis through integrin alphavbeta8-mediated activation of TGF-beta. J Clin Invest..

[18] Araya J (2007). Squamous metaplasia amplifies pathologic epithelial-mesenchymal interactions in COPD patients. J Clin Invest..

[19] Coraux C, Hajj R, Lesimple P, Puchelle E (2005). *In vivo* models of human airway epithelium repair and regeneration. Eur Respir Rev..

[20] Longmire TA (2012). Efficient derivation of purified lung and thyroid progenitors from embryonic stem cells. Cell Stem Cell..

[21] Wong AP, Rossant J (2013). Generation of Lung Epithelium from Pluripotent Stem Cells. Curr Pathol Biol Rep..

[22] Ghaedi M (2013). Human iPS cell-derived alveolar epithelium repopulates lung extracellular matrix. J Clin Invest..

[23] Ghaedi M (2014). Alveolar epithelial differentiation of human induced pluripotent stem cells in a rotating bioreactor. Biomaterials..

[24] Zuo W (2015). p63(+)Krt5(+) distal airway stem cells are essential for lung regeneration. Nat..

[25] Vaughan AE (2015). Lineage-negative progenitors mobilize to regenerate lung epithelium after major injury. Nat..

[26] Kardia, E., Ch’ng, E. & Yahaya, B. Aerosol‐based Airway Epithelial Cell Delivery Improves Airway Regeneration and Repair. *J Tissue Eng Regen Med*. (2017). 10.1002/term.2421 28105760

[27] Dosanjh A, Morris RE, Wan B (2001). Bronchial epithelial cell-derived cytokine IL-10 and lung fibroblast proliferation. Transplant Proc..

[28] Fiorentino DF, Zlotnik A, Mosmann TR, Howard M, O’Garra A (2016). Pillars Article: IL-10 Inhibits Cytokine Production by Activated Macrophages. J. Immunol. 1991. 147: 3815-3822. J Immunol..

[29] Seitz M (1995). Interleukin-10 differentially regulates cytokine inhibitor and chemokine release from blood mononuclear cells and fibroblasts. Eur J Immunol..

[30] Hong KU, Reynolds SD, Watkins S, Fuchs E, Stripp BR (2004). *In vivo* differentiation potential of tracheal basal cells: evidence for multipotent and unipotent subpopulations. Am J Physiol Lung Cell Mol Physiol..

[31] Rock JR (2009). Basal cells as stem cells of the mouse trachea and human airway epithelium. PNAS..

[32] Tata PR (2013). Dedifferentiation of committed epithelial cells into stem cells *in vivo*. Nat..

[33] Rawlins EL, Ostrowski LE, Randell SH, Hogan BL (2007). Lung development and repair: contribution of the ciliated lineage. PNAS..

[34] Kanzaki M (2006). Tissue engineered epithelial cell sheets for the creation of a bioartificial trachea. Tissue Eng..

[35] Kojima K (2003). A composite tissue-engineered trachea using sheep nasal chondrocyte and epithelial cells. FASEB J..

[36] Hajj R (2007). Basal cells of the human adult airway surface epithelium retain transit-amplifying cell properties. Stem Cells..

[37] Blanpain C, Horsley V, Fuchs E (2007). Epithelial stem cells: turning over new leaves. Cell..

[38] Frank DB (2016). Emergence of a Wave of Wnt Signaling that Regulates Lung Alveologenesis by Controlling Epithelial Self-Renewal and Differentiation. Cell Rep..

[39] Okubo T, Hogan BL (2004). Hyperactive Wnt signaling changes the developmental potential of embryonic lung endoderm. J Biol..

[40] Cohen ED (2009). Wnt signaling regulates smooth muscle precursor development in the mouse lung via a tenascin C/PDGFR pathway. J Clin Invest..

[41] Goss AM (2009). Wnt2/2b and beta-catenin signaling are necessary and sufficient to specify lung progenitors in the foregut. Dev Cell..

[42] Hogan BL (2014). Repair and regeneration of the respiratory system: complexity, plasticity, and mechanisms of lung stem cell function. Cell Stem Cell..

[43] Barkauskas CE (2013). Type 2 alveolar cells are stem cells in adult lung. J Clin Invest..

[44] Prewitz MC (2013). Tightly anchored tissue-mimetic matrices as instructive stem cell microenvironments. Nat. Methods..

[45] Ellis SJ, Tanentzapf G (2010). Integrin-mediated adhesion and stem-cell-niche interactions. Cell Tissue Res..

[46] Volckaert T, De Langhe S (2014). Lung epithelial stem cells and their niches: Fgf10 takes center stage. Fibrogenesis Tissue Repair..

[47] Puchelle E, Zahm JM, Tournier JM, Coraux C (2006). Airway epithelial repair, regeneration, and remodeling after injury in chronic obstructive pulmonary disease. Proc Am Thor Soc..

[48] Mura M, dos Santos CC, Stewart D, Liu M (2004). Vascular endothelial growth factor and related molecules in acute lung injury. J Appl Physiol..

[49] Ali M, Lillehoj EP, Park Y, Kyo Y, Kim KC (2011). Analysis of the proteome of human airway epithelial secretions. Proteome Sci..

[50] Sohal SS (2010). Reticular basement membrane fragmentation and potential epithelial mesenchymal transition is exaggerated in the airways of smokers with chronic obstructive pulmonary disease. Respirology..

[51] Pakyari M, Farrokhi A, Maharlooei MK, Ghahary A (2013). Critical Role of Transforming Growth Factor Beta in Different Phases of Wound Healing. Adv Wound Care..

[52] Halwani R, Al-Muhsen S, Al-Jahdali H, Hamid Q (2011). Role of transforming growth factor-beta in airway remodeling in asthma. Am J Respir Cell Mol Biol..

[53] Kumar RK, Herbert C, Foster PS (2004). Expression of growth factors by airway epithelial cells in a model of chronic asthma: regulation and relationship to subepithelial fibrosis. Clin Exp Allergy..

[54] Kardia, E., Halim, N. S. & Yahaya, B. H. Aerosol-Based Cell Therapy for Treatment of Lung Diseases. *Methods Mol Biol*. 1–13 (2016). 10.1007/7651_2016_327 27062596

