# Supplementary material for: Stimulatory Secretions of Airway Epithelial Cells Accelerate Early Repair of Tracheal Epithelium
Source: Sci Rep. 2017 Sep 15;7:11732. doi: 10.1038/s41598-017-11992-6 (PMC5601923; doi:10.1038/s41598-017-11992-6)
Supplement: Supplementary file 1 — Supplementary Information [file 41598_2017_11992_MOESM1_ESM.pdf]

Stimulatory Secretions of Airway Epithelial Cells Accelerates Early Repair of Tracheal Epithelium

Egi Kardia, Rafeezul Mohamed, and Badrul Hisham Yahaya

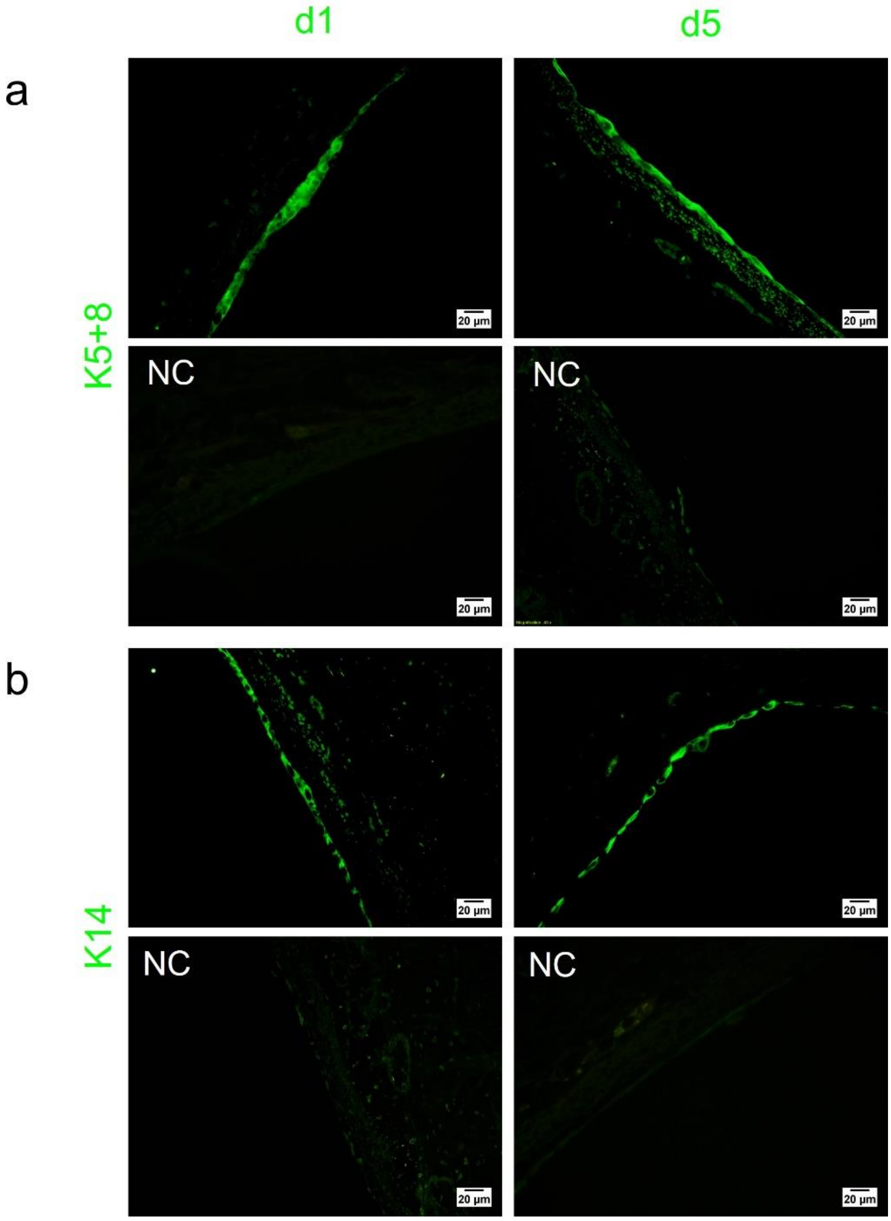

Supplementary figure 1 Representative images of (a) K5+8 (green) and (b) K14 (green) staining of seeded AECs in the damaged tracheal basement membrane at day 1 and 5 following direct co-culture assay. The negative control samples (NC) were given the similar staining without primary antibody incubation (scale bars: 20 µm).

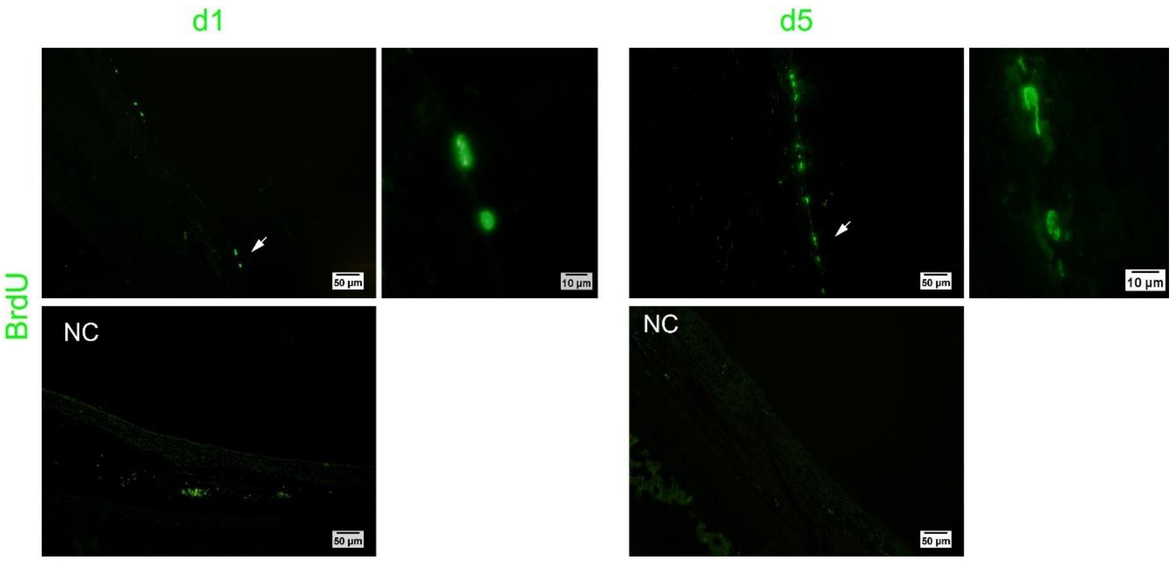

Supplementary figure 2 Representative images of BrdU (green) staining of seeded AECs in the damaged tracheal basement membrane at day 1 and 5 following direct co-culture assay. Arrows indicate BrdU^{+} cells that formed the lining of epithelium. The negative control samples (NC) were given the similar staining without primary antibody incubation (scale bars: 50 μm, scale bars of inserts represent 10 μm).

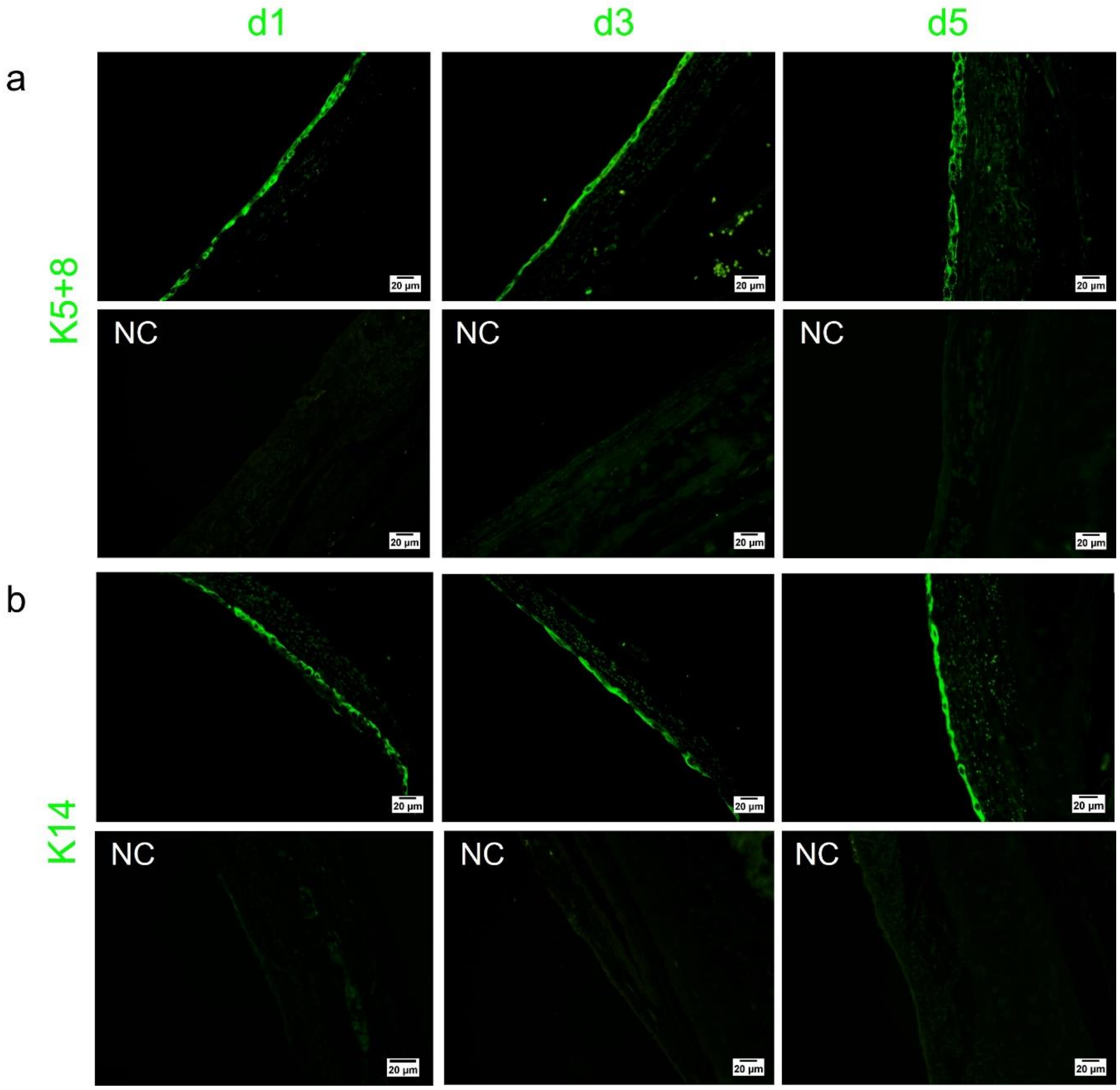

Supplementary figure 3 Representative images of (a) K5+8 (green) and (b) K14 (green) staining of tracheal epithelium following indirect AEC co-culture assay at day 1, 3, and 5. The negative control samples (NC) were given the similar staining without primary antibody incubation (scale bars: 20 µm).

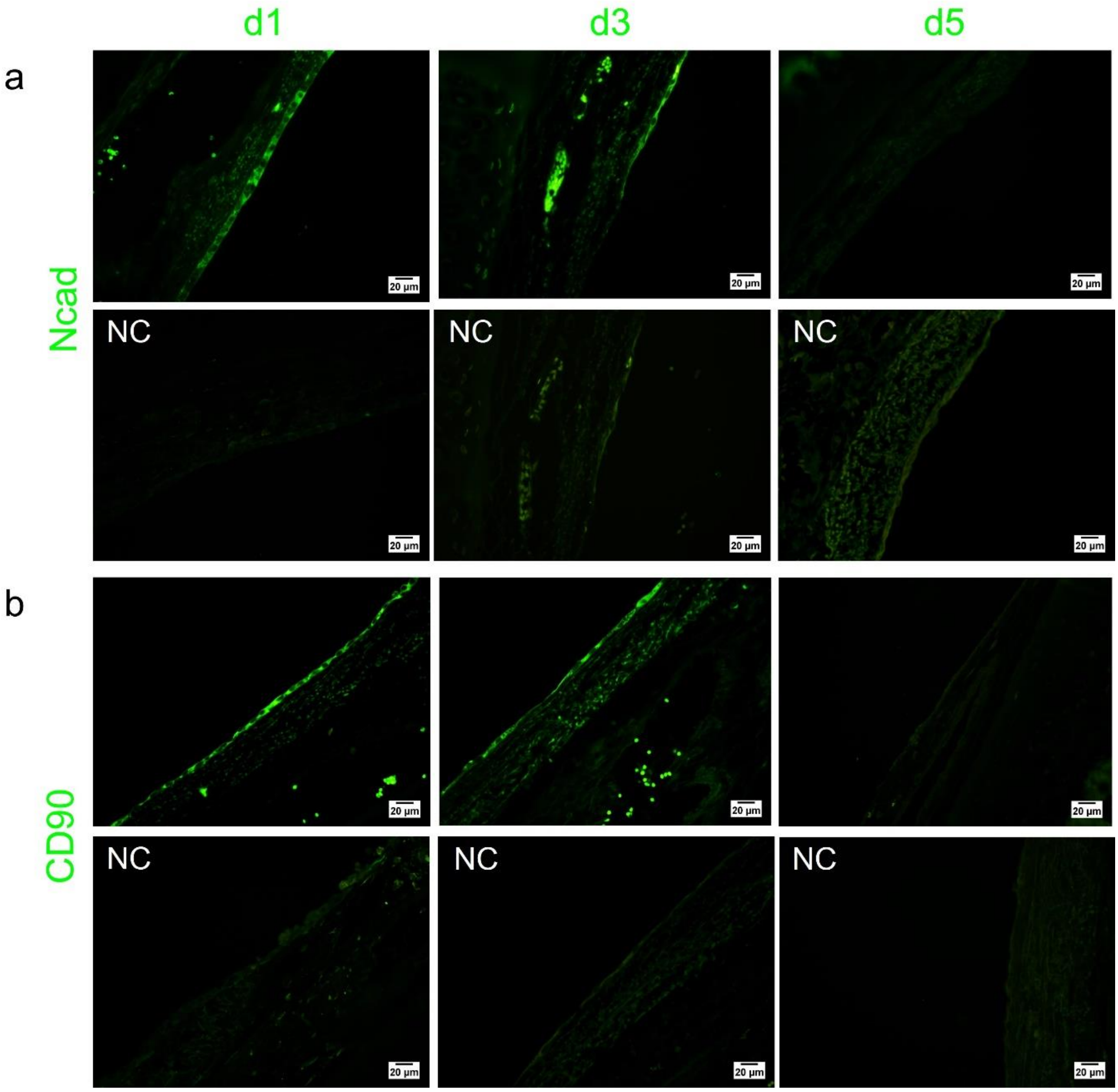

Supplementary Figure 4 Representative images of (a) N-cadherin (green) and (b) CD90 (green) staining of tracheal epithelium following indirect AEC co-culture assay at day 1, 3, and 5. The negative control samples (NC) were given the similar staining without primary antibody incubation (scale bars: 20 µm).
